# MTA1 promotes nasopharyngeal carcinoma growth *in vitro* and *in vivo*

**DOI:** 10.1186/1756-9966-32-54

**Published:** 2013-08-14

**Authors:** Qingcui Song, Hong Zhang, Min Wang, Wen Song, Min Ying, Yuan Fang, Yiyi Li, Yilan Chao, Xiaoxia Zhu

**Affiliations:** 1 Cancer Research Institute, Key Lab for Transcriptomics and Proteomics of Human Fatal Diseases, Nanfang Hospital, Southern Medical University, Guangzhou, Guangdong Province, China; 2 Department of Radiation Oncology, Nanfang Hospital, Southern Medical University, Guangzhou, Guangdong Province, China

**Keywords:** MTA1, Nasopharyngeal carcinoma, Cell proliferation, Tumorigenesis

## Abstract

**Background:**

The prognostic value of metastasis-associated gene 1 (MTA1) in nasopharyngeal carcinoma (NPC) has been suggested. However, there is still no direct evidence that MTA1 promotes NPC growth in vivo. In this study, we aimed to investigate the function of MTA1 in the regulation of NPC cell proliferation and tumorigenesis *in vitro* and *in vivo*.

**Methods:**

Stable MTA1 knockdown or overexpression NPC cell lines were employed. The effects of MTA1 depletion or overexpression on cell proliferation, colony formation, cell cycle progression were examined by MTT, colony formation and flow cytometry assay. The effects of MTA1 depletion on tumor growth in vivo were examined in mouse xenograft model.

**Results:**

*MTA1* knockdown or overexpression drastically changed the proliferation, colony formation and cell cycle of NPC cells *in vitro. MTA1* depletion significantly suppressed NPC tumorigenesis *in vivo*.

**Conclusion:**

MTA1 promotes NPC cell proliferation via enhancing G1 to S phase transition, leading to increased tumor growth. Targeting MTA1 is a promising approach to reduce tumor burden of NPC.

## Introduction

Nasopharyngeal carcinoma (NPC) is an epithelial malignant tumor with a high incidence in southern China and Southeast Asia. Radiotherapy is a dominant treatment approach for NPC. Primary tumor volume (GTV-P) is known to be positively correlated with the prognosis of NPC [1,2]. Despite recently increased use of intensity-modulated radiation therapy (IMRT), GTV-P is still an independent prognostic indicator for treatment outcome of NPC, and has correlations with T classification, cervical lymph node metastasis as well as post-treatment distant metastasis [3,4]. Tumor volume is known to be positively correlated with the proliferation ability of tumor cells. Thus further understanding of molecular mechanisms underlying abnormal proliferation of NPC cells will help develop novel options for the diagnosis, therapy and prognosis of NPC.

Metastasis-associated gene 1 (MTA1) has been implicated in the carcinogenesis and metastasis of a variety of human cancers [5-7]. In particular, recent studies suggest the prognostic value of MTA1 in NPC because MTA1 overexpression was an independent prognostic factor for poor overall survival of NPC patients [8,9]. Our recent study provided direct evidence that MTA1 regulated actin cytoskeleton reorganization to promote NPC metastasis [7]. However, the role of MTA1 in NPC cell proliferation is not clear.

In the present study, we employed both gain and loss of function approaches to investigate the role of MTA1 in NPC growth. We examined the effects of MTA1 overexpression or knockdown on NPC cell proliferation, cell-cycle distribution, and colony formation *in vitro*. In addition, we evaluated the effects of MTA1 knockdown on NPC xenograft growth in nude mice. Our results showed that MTA1 promoted NPC growth in vitro and in vivo.

## Methods

### Cell culture

Stable *MTA1* knockdown NPC cell lines (CNE1/MTA1-si and C666-1/MTA1-si), stable *MTA1* overexpression NPC cell line (NP69/MTA1), and their corresponding control cells (CNE1 or C666-1/CTL-si, and CNE1, C666-1 or NP69/NC) were constructed and cultured as described in previous study [7]. CNE1 were well-differentiated NPC cells, C666-1 were undifferentiated NPC cells, and NP-69 were immortalized NPC cells.

### Cell proliferation assay

The cells were plated into 96-well plates at the density of 5,000 cells/well and cultured in RPMI-1640 medium supplemented with 10% fetal bovine serum for 1, 2, 3, 4, 5, 6 and 7 days, respectively. Then cells were incubated with 20 μL MTT [3-(4, 5-dimethylthiazol-2-yl)-2, 5-diphenyl tetrazolium bromide] (5 mg/mL) (Promega, Shanghai, China) for additional 4 h, and 100 μL DMSO was added into each well to dissolve the formazan product. The absorbance of the enzyme was measured at 490 nm using an Microplate Reader (Bio-Rad, Hercules, CA, USA). Cell growth rates (average absorbance of each group) were then calculated. All experiments were performed in triplicate samples and repeated at least three times.

### Colony formation assay

The cells were grown in 6-well plates and cultured in a humidified incubator at 37°C and 5% CO_2_. The cells were then continuously cultured until visible colonies were formed (14 days). The colonies were fixed with methanol for 15 min, stained with hematoxylin for 10–15 min, and colonies containing >50 cells were counted. The rate of colony formation was indicated by the ratio of the number of colonies over the number of seeded cells. The experiment was repeated three times, and a mean value was presented.

### Cell cycle analysis

Cell cycle distribution was detected by using Cycletest Plus DNA Reagent kit (Becton Dickinson, USA). The protocol recommended by BD Bioscience was followed. The samples were run with a FACScan flow cytometer (Becton Dickinson, USA) and the results obtained were analyzed using the ModFit software.

### Xenograft model

Female athymic BALB/c nu/nu mice (4–6 weeks old) were purchased from Guangdong Medical Laboratory Animal Center (Guangzhou, China). All protocols for animal studies were reviewed and approved by Animal Care and Use Committee of Southern Medical University. 1 × 10^7^ cells from individual cloned cell lines were injected subcutaneously into the left flank and right flank of nude mice (n = 5 per group). After 10 days of implantation of tumor cells, tumors were measured with calipers every 3 days. Tumor volume was calculated according to the following formula: V = (L*W^2^*π)/6, V, volume (mm^3^); L, biggest diameter (mm); W, smallest diameter (mm) [10]. At the end of experiments, the mice were euthanized and tumors were excised and weighed.

### Immunohistochemical staining

Immunohistochemical staining was performed using standard protocol. Briefly, cryosections were stained with primary antibodies for MTA1 (1:200; Abcam, USA) and Ki67 (1:200; Abcam, USA), followed by incubation with secondary antibodies. A stained cell was considered as positive cell. All results of immunohistochemical staining were double-blinded judged by different pathologists.

### Statistical analysis

All data were presented as the mean ± standard deviation of at least three independent experiments. The two-tailed unpaired Student’s *t* test was used to assess differences in cell growth rate, colony formation, cell cycle distribution, tumor weight, tumor volume and immunohistochemistry stained cell count between groups. *P* < 0.05 was considered statistically significant.

## Results

### *MTA1* regulates NPC cell growth *in vitro*

**Figure 1:**
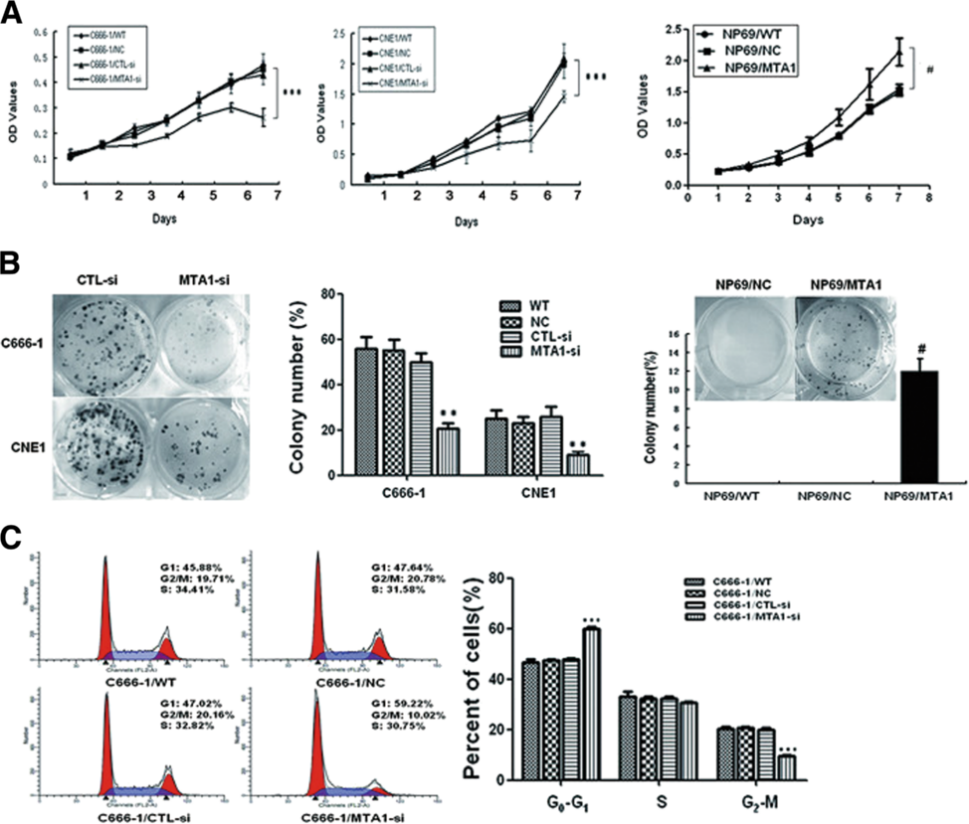
***MTA1 *promotes the growth of NPC cells *in vitro*. (A)** MTT proliferation assay of *MTA1* knockdown cell lines, *MTA1* overexpression cell lines and control cells. **(B)** Representative images of colony formation assay of *MTA1* knockdown cell lines, *MTA1* overexpression cell lines and control cells. **(C)** Flow cytometry analysis of cell-cycle distribution of *MTA1* knockdown C666-1 cells and control cells. All results were reproducible in three independent experiments. CTL-si versus WT: P > 0.05; **P < 0.01, ***P < 0.001 compared to CTL-si. # P < 0.001 compared to NC. OD, optical density.

First we examined the effect of endogenous *MTA1* knockdown on NPC cell growth. MTT assay showed that *MTA1* knockdown reduced the cell growth rate by 44% in C666 cells (*P* < 0.001) and by 30% in CNE1 cells (*P* < 0.001) (Figure 1A). Colony formation assay showed that *MTA1* knockdown resulted in dramatic decrease of colony-formation efficiency in C666-1 and CNE1 cells, compared to their corresponding controls (*P* <0.01; Figure 1B). These data imply that endogenous *MTA1* is essential to the proliferation and colony formation of NPC cells.

To further investigate the function of *MTA1* in NPC cell growth, we performed gain-of-function experiments in immortalized nasopharyngeal epithelial cell NP69. Compared with the cells transfected with empty vector, enforced *MTA1* overexpression significantly promoted the growth and colony-formation capacity of NP69 cells (p < 0.001; Figure 1A and B).

To understand how *MTA1* promotes NPC cell proliferation and colony formation, we examined cell cycle progression of C666-1 cells depleted of *MTA1*. Compared with control cells, C666-1/MTA1-si cells displayed an increased percentage of cells in G1 phase and fewer cells in G2 phase (p < 0.001), but no significant difference in S phrase distribution (Figure 1C). The results demonstrate that *MTA1* knockdown induced cell cycle arrest at G1.

### *MTA1* depletion inhibits the growth of NPC xenografts *in vivo*

**Figure 2:**
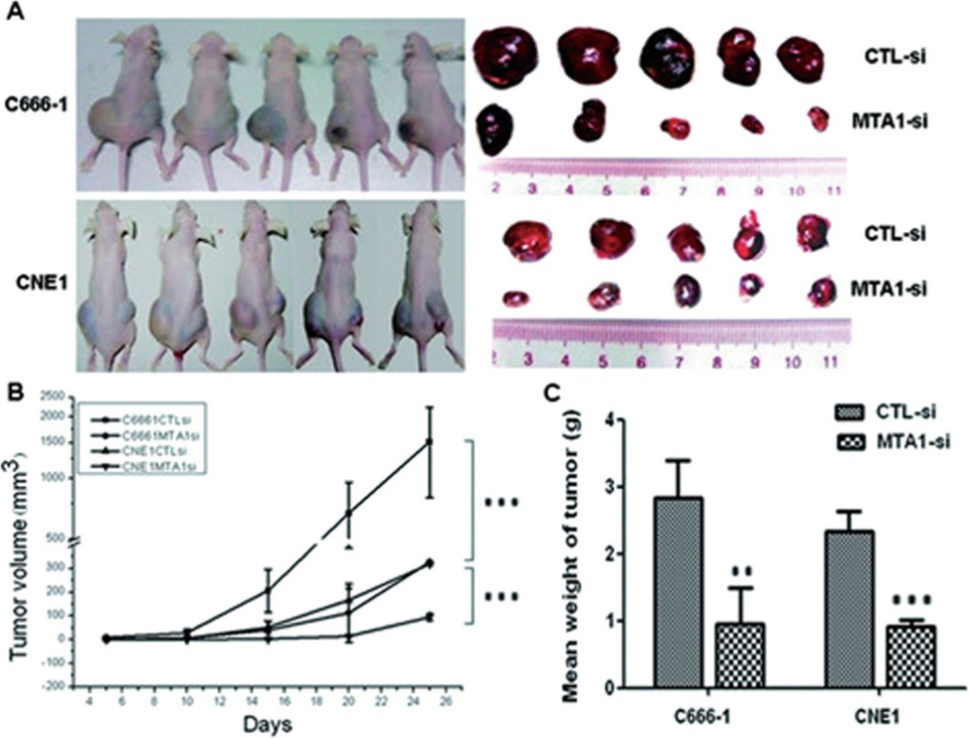
***MTA1 *depletion inhibits NPC tumorigenesis *in vivo*. (A)***MTA1* knockdown NPC cells were injected subcutaneously into the right flank of nude mice. Control cells were injected subcutaneously into the left flank of the same nude mice (n = 5). At 3 weeks after implantation, *MTA1* knockdown cells produced smaller tumors than control cells. **(B)** Growth curve of tumor volumes. Each data point represented mean ± SD of 5 mice. **(C)** The tumor from each group was weighed immediately after the dissection. The average tumor weight was indicated as mean ± SD. **P < 0.01, ***P < 0.001 as compared to CTL-si.

To assess the effect of *MTA1* on NPC growth *in vivo*, we injected *MTA1* depleted C666-1 or CNE1 cells, or their control cells into nude mice subcutaneously, and then monitored tumor growth. Palpable tumors were first detected in all mice by day 10 after injection. At the end of experiments, all the mice developed tumors (Figure 2A). Compared with control, the average tumor volume in mice injected with *MTA1* depleted C666-1 or CNE1 cells was markedly reduced by more than 60% (p < 0.01; Figure 2B). The average tumor weight was also significantly reduced in *MTA1* depleted group (p < 0.01; Figure 2C).

**Figure 3:**
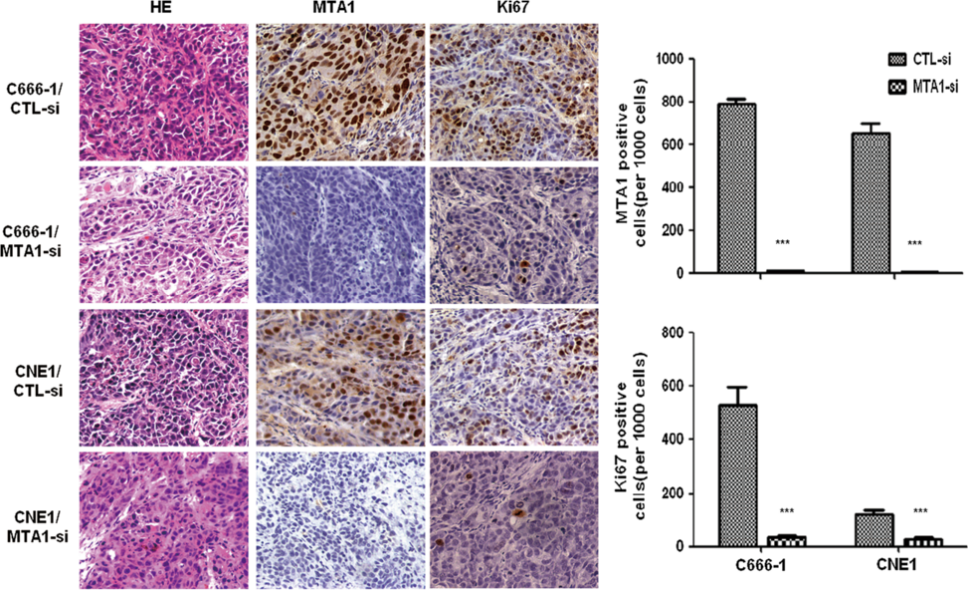
**Immunohistochemistry staining of Ki67 in mouse xenograft models.** MTA1 and Ki67 staining was less in subcutaneous tumor tissues derived from *MTA1* knockdown NPC cells, compared with those from control cells (Magnification, ×200).

Further immunohistochemical assessment of the nuclear antigen Ki-67 was used to estimate cell proliferation. The results demonstrated that the number of Ki-67 positive cells was significantly decreased in tumor nodules originating from *MTA1* depleted cells, compared to control cells (Figure 3).

## Discussion

MTA1 has been shown to be overexpressed in human cancers [5]. However, the clinicopathological evidence to support the correlation of MTA1 overexpression with tumor growth is limited. Only one report demonstrated that MTA1 overexpression was associated with larger tumor size in hepatocellular cancer [11]. Several studies examined the clinicopathological significance of MTA1 in NPC, but found no association between increased MTA1 expression and T-stage [8,9]. This may be due to the limitations of current T staging system of NPC for determining tumor burden [3]. The inclusion of tumor volume into TNM staging system has been proposed [3,4]. Thus the biological relevance of MTA1 to NPC growth and tumor volume need to be further investigated.

In fact, MTA1 is clearly involved in breast cancer growth. Antisense of MTA1 inhibited the growth of highly metastatic breast cancer cell lines [12]. Moreover, forced expression of MTA1 nhanced the ability of breast cancer cell line MCF-7 to grow in an anchorage-independent manner [13]. MTA1 controbutes to inappropriate development of mammary glands, hyperplastic nodules and mammary tumors [14,15]. In our study, we transfected MTA1 cDNA into immortalized nasopharyngeal epithelial cell and showed that enforced expression of MTA1 contributed to increased cell growth and colony formation, consistent with the results by Mahoney et al. [16]. We further examined the therapeutic value of MTA1 siRNA and found that downregulation of MTA1 by RNAi successfully suppressed the growth of C666-1 NPC cells *in vitro* and *in vivo*, suggested that MTA1 is a promising target for NPC gene therapy.

The abnormal cell cycle is crucial to the proliferation of cancer cells. Different from our findings in lung cancer cells [17], in the present study, we provided evidence that MTA1 knockdown induced G1 arrest of NPC cells, suggesting that MTA1 promotes aberrant G1 to S phase transition, leading to increased proliferation and tumorigenicity of NPC cells. These divergent findings suggest that the effect of MTA1 on tumor cell growth and cell cycle progression are cell dependent. Cell cycle is regulated by a variety of signaling pathways, among which p53 pathway is a crucial regulator of cell cycle and apoptosis of cancer cells [18]. Emerging data suggest that MTA1 had deacetylation activity on p53 and subsequently attenuated the transactivation function of p53 [19,20]. MTA1 was also identified as a p53-independent transcriptional corepressor of p21 (WAF1), which is a direct target of p53 and mediates p53-dependent G1 growth arrest [21].

## Conclusions

In summary, we found that MTA1 knockdown in NPC cells decreases cell proliferation *in vitro* via the induction of G1 phase arrest and drastically suppresses tumor formation *in vivo*. These findings suggest that targeting MTA1 is a promising approach to reduce tumor burden of NPC.

## Competing interest

The authors declare that they have no competing interests.

## Authors’ contributions

QS, HZ and MW carried out the in vitro experiments. WS, MY and YF carried out the in vivo experiments. YL and YC performed statistical analysis. XZ conceived of the study, participated in its design and coordination and drafted the manuscript. All authors read and approved the final manuscript.
